# ‘I have used up my entire youth in the bush’: the *Comités de Autodefensa* during and after the Peruvian internal armed conflict

**DOI:** 10.1007/s10611-022-10049-8

**Published:** 2022-10-05

**Authors:** Eva Willems

**Affiliations:** Center for Conflict Studies, Philipps-University of Marburg, Marburg, Germany

**Keywords:** Self-defense militias, Civilian participation in armed conflict, Citizenship, Disarmament demobilization and reintegration (DDR), Transitional justice (TJ), Peru

## Abstract

Youngsters participate as combatants at the forefront of armed conflicts around the globe, be it as part of state forces, as members of rebel groups, or as drivers of armed civilian resistance. This contribution explores the social trajectories of (ex-)civil self-defense militia members in Peru who fought alongside the state forces to defeat the Maoist rebels of Shining Path in the 1980 and 1990s. On the one hand, by taking the Peruvian *Comités de Autodefensa* (CAD) as a somewhat atypical case-study, the article aims to enhance a more nuanced understanding of youth as drivers of and participants in civil war violence which transcends the victim-perpetrator dichotomy. On the other, by analyzing the social trajectories of CAD leaders and members from their youth until the present, it seeks to gain insight into ex-combatants’ claims for recognition, reparation and citizenship in the aftermath of armed conflict. The trajectories of the CAD members demonstrate how the morality of soldiering, steered by ideas about masculinity, militarism and patriotism, gets intertwined with structural societal conditions such as the lack of educational and economic perspectives for youngsters, and the state’s failure to provide protection and security against rebel group violence to those who might need it most. In the aftermath of the conflict, militia service and the corresponding *macho* warrior identity form a basis of demands for inclusion by an historically marginalized rural population group. The findings on the Peruvian self-defense committees presented in this article have several implications for research and policy in the fields of Transitional Justice and Disarmament, Demobilization and Reintegration, and open both thematic and conceptual avenues for further research into civilian participation in armed conflicts around the globe.

## Introduction

In the valley of the Apurímac River^1^ in the region of Ayacucho, Peru, the *Comités de Autodefensa* (CAD) or self-defense committees emerged as an important additional actor involved in the internal armed conflict between the Maoist rebels of Shining Path and the state forces during the 1980 and 1990s.^2^ These self-defense committees best fit the definition of militias provided by Jentzsch, Kalyvas and Schubiger: ‘armed groups that operate alongside regular security forces or work independently of the state to shield the local population from insurgents’ (Jentzsch et al., 2015, p. 755). As the authors point out, while other definitions have used the term militias to describe any nonstate armed groups, the crucial element in this definition is the anti-rebel dimension. Indeed, while peasant communities in the Apurímac valley initially founded the CADs as a means to protect themselves from violent attacks committed by both the state forces and Shining Path, the self-defense committees eventually played a crucial role in fighting alongside the army to defeat the Maoist rebels. After the end of the internal armed conflict, the CADs of the Apurímac valley were never actively dismantled nor disarmed. As the region is characterized by protracted conflict between the state forces and former Shining Path rebels turned criminal drug clans, the CADs in many communities keep on functioning as local security forces.

While civil self-defense militias have emerged in many conflicts, they rarely are the object of study, as the proliferation and fragmentation of armed actors is mostly studied from a rebel perspective (Jentzsch et al., 2015, p. 756). Accordingly, fields concerned with reconstruction after armed conflict, such as peacebuilding, transitional justice (TJ) or demobilization, disarmament and reintegration (DDR), tend to see a dichotomous post-conflict world populated by civilians and (ex-)combatants, or victims and perpetrators (Willems, 2020). Both in conflict and post-conflict studies, civilian participation in wartime violence therefore still lacks nuance and further conceptualization – a claim which also holds true for the Peruvian case. As an armed actor in need of asserting its authority, the civil self-defense committees contributed to the proliferation of violence and human rights violations during the internal armed conflict.^3^ According to the Truth and Reconciliation Commission (*Comisión de la Verdad y la Reconciliación*, CVR) which investigated the events of twenty years of civil war violence during Peru’s transition to democracy (2001–2003),

[…] for no other actor of the war, the dividing line between perpetrator and victim, between hero and villain, is so thin and porous as for the self-defense committees. As pacifiers for some and murderers for others, they are a concern for all […] (CVR, 2003, p. 74)

The present article aims to contribute to a better understanding of civilian participation in armed conflict by exploring the social trajectories of (ex-)CAD combatants in the Apurímac valley from their youth until the present. Children and youngsters participate as combatants at the forefront of internal and international armed conflicts around the globe. Existing scholarship has engaged with the recruitment - whether forced or voluntary - of children and youth into rebel groups or state armies, as well as the demobilization of child soldiers and their reintegration into society (see, for example, Machel, 2001). In the Latin American context, youngsters’ participation in armed gang violence has furthermore received particular attention (see, for example, Rodgers & Baird, 2015).

The role of youth as drivers of armed civilian resistance against rebel group and state perpetrated violence, however, remains largely under-investigated. This perspective nevertheless seems relevant for studying self-defense militias such as the Peruvian CADs, given the fact that youngsters between the age of sixteen and twenty-five made up the bulk of the combatants and took up central roles. One of the mythical leaders of the self-defense committees in the Apurímac valley, Antonio Cárdenas, was only nineteen years old when he founded the headquarters of the regional CAD network in the town of Pichiwillca in 1984. The militarization of daily life during crucial years of their youth severely influenced the (ex-)CAD leaders’ and members’ aspirations for the present and future. Today, as they try to cope with the physical and mental injuries caused by the violence of the internal armed conflict, many feel that both their past and future were ‘stolen’ by the war. At the same time, they are confronted with a present-day reality characterized by structural conditions of poverty and exclusion that did not only form the initial breeding ground for the internal armed conflict — as was concluded by the CVR in its final report —but now also thwart the ex-combatants’ personal development, as well as that of their children and grandchildren.

Following Bourdieu, youth in the first place constitutes a social construct as ‘the divisions between the ages are arbitrary’ (1993, p. 94), and arguably even more so during times of war and crisis, when social relations get disrupted beyond average. For the purpose of the present article, youth actors can be considered those who at the start of the internal armed conflict in 1980 were “old enough” to play their part in wartime violence, but “young enough” to outlive the conflict and foster expectations for a new beginning during the aftermath. Adopting a social trajectory lens to study the role of youth actors in armed conflict, then, allows for an analysis that integrates past, present and future motivations, actions and desires. Looking at the phenomenon of armed civilian resistance against state and rebel group violence through said lens will serve two interconnected objectives. On the one hand, by taking the Peruvian self-defense committees as an atypical case-study, the article aims to enhance a more nuanced understanding of youth as drivers of and participants in civil war violence which transcends the victim-perpetrator dichotomy and sketches a more complex picture of youngsters’ motivations to become combatants. On the other, by analyzing the trajectories of CAD leaders and members from their youth until the present, it seeks to gain insight into ex-combatants’ claims for recognition, reparation and citizenship in the aftermath of armed conflict.

In order to achieve these objectives, I will first focus on the social trajectories of CAD combatants during the internal armed conflict to investigate the motivations of youngsters to organize and participate in armed resistance against violence perpetrated by Shining Path and the state forces. I will hereby shed light on the general structures of organization and recruitment of the CADs during the internal armed conflict, and look at the role of gender and masculinities in constructing identities within the self-defense committees. Subsequently, I will turn to the CADs’ present-day discourses on recognition and citizenship, and argue that their feelings of loss on a personal level go hand in hand with a sense of being abandoned by the state, and a desire for inclusion into the nation.

A better understanding of civilian participation in armed conflict along the lines of the article’s two abovementioned objectives can advance research and policy in the fields of TJ as well as DDR. Both fields share many of their long-term goals but have nevertheless developed mostly separate from each other due to their different beneficiaries, respectively victims and perpetrators (Waldorf, 2009, p. 16; Theidon, 2009, p. 2). Firstly, going beyond this victim-perpetrator binary by studying the CADs as actors with a complex social trajectory, steered by both ideological and pragmatic decision-making processes, allows us to better understand how structural conditions such as socio-economic exclusion did not only shape the conflict, but also continue to define the daily post-conflict reality. Secondly, the social trajectories of the CADs reveal how notions of masculinity and heroism play out in processes of meaning-making, collective memory and identity construction among (ex-)combatants, both during and in the aftermath of armed conflict. While these notions are often at odds with human rights and victimhood discourses that guide post-conflict reconstruction efforts, they can as well —as the case-study of the Peruvian CADs shows — be deployed by survivors to formulate claims for recognition, reparation and citizenship that align with the goals of TJ and DDR.

In what follows, before turning to the analysis of past and present trajectories of civil self-defense committees in Peru, I will first briefly outline my methodology, as well as the historical background against which the CADs emerged.

## Methodology

The findings in this article are based on interviews and participant observation with (ex-)civil self-defense militia members during several fieldtrips in the Apurímac valley conducted between 2015 and 2021 on the one hand, and sources found in the archive of the central headquarter of the CADs of the Apurímac valley in the town of Pichiwillca on the other.

More specifically, together with, Gabriela Zamora (historian of the *Universidad Nacional San Cristobal de Huamanga* in Ayacucho), we conducted forty interviews in twenty small villages *(centros poblados)* in the districts of Samugari and Santa Rosa (La Mar, Ayacucho), Kimbiri and Pichari (La Convención, Cusco), Llochegua (Huanta, Ayacucho), and Rio Tambo (Satipo, Junín). Of the forty interviewees, thirty-two are ex-CAD members – meaning that they participated in one way or another in self-defense – of whom thirteen are ex-commanders who took up leading roles. Other interviewees include relatives of ex-CADs, actual CAD members and local authorities. The fact that the fieldwork was multi-sited reflects the nature of the object of study: the CADs were organized in a network that covered the entire valley, and we followed the structure of their organization for our fieldwork. Potential interviewees were initially selected through our previous knowledge of the subject which made it possible to identify key informants, and subsequently through snowball sampling. Participant observation was carried out during the commemoration ceremony of the 34th anniversary of the foundation of the self-defense committees of the valley in the village of Pichiwillca in June 2018, during several town meetings and patron saint celebrations, and during a training of the current CAD by the military in the district of Samugari.

In addition to the interviews and participant observation, the findings presented in this article build upon archival sources produced between 1976 and 2003 which are kept at the central headquarter of the self-defense committees of the Apurímac valley in the town of Pichiwillca. The types of documents include, among others, correspondence between the base committees and the headquarter concerning military operations and daily governance; testimonies of prisoners captured by the self-defense committees; communication between the self-defense committees and the Peruvian army; and certificates and permissions granted by the headquarter of the self-defense committee to community members.^4^

In order to guarantee the privacy of the research participants, I refer to the districts but not to the specific villages or towns where the fieldwork was conducted. All names of interviewees are pseudonyms. As for the information found in the archival documents, no sensitive personal data is disclosed. In addition to informed consent in case of the interviews, general permission to carry out the field and archival research was granted by the current leadership of the self-defense committee of Pichiwillca.

## The *Comités de Autodefensa* in the Apurímac valley

The Apurímac valley – today part of the larger geopolitical region of the valley of the Rivers Apurímac, Ene and Mantaro (commonly abbreviated as VRAEM) – has a particular history of indigenous inhabitation, colonization and armed struggle. In the seventeenth century, Jesuits and Franciscans made their way into the cloud forest, eager to Christianize the native Asháninka population. At the beginning of the twentieth century, settlers *(colonos)* from the Northern highlands of Ayacucho entered the valley in search for better lands and lives (Del Pino, 1996, p. 123–128). From the 1950s onwards, the region became home to farmers who started growing cocoa, coca, coffee, peanuts and *barbasco*^5^. Between 1968 and 1975, the agricultural reforms of president Velasco Alvarado obliged the redistribution of the land of the *hacendados*, which gave rise to *minifundias*^6^ and small cooperatives of farmers (Heuser, 2017, p. 191–194). As the *colonos* almost exclusively came from the Northern highlands of Ayacucho and maintained strong ties with their communities of origin, they imported the Andes culture into the subtropical valley. As a response to the invading settlers, the native Asháninka population was largely displaced downstream towards the valley of the River Ene. Until today, however, several Asháninka communities in the Apurímac valley coexist with the *colonos* from the highlands, although their territory was fiercely reduced by the settlers (CVR, 2003, p. 74).

As soon as Shining Path initiated its armed revolution in the highlands of Ayacucho in 1980, more peasants displaced by the violence sought refuge in the Apurímac valley. The region was then still considered a safe zone, but it would not take long before Shining Path would make its way into the forest. Between 1980 and 1982, the rebels appeared several times, mostly intimidating or killing existing authorities. The violent escalation of the war in the Apurímac valley fully took off in 1983. In response, the army’s marine forces established bases throughout the region and started a particularly violent and repressive counteroffensive (CVR, 2003, p. 84).

Persecuted by the war, peasants decided to organize against the violence perpetrated by both the state forces and Shining Path. The first self-defense committees, the so-called *montoneros*, organized in the Southern part of the valley, in the highland districts of Chungui and Anco (Fumerton, 2002, p. 115).^7^ In 1984, these *montoneros* held a march downstream from Chungui to San Francisco and Kimbiri in order to encourage peasant settlements and communities throughout the valley to organize in self-defense committees. The idea quickly gained ground and the previous organizational experience of peasant cooperatives helped to bring about an efficient regional network of self-defense.

The main headquarter of the self-defense committees of the valley *-* the *Sede Central del Comité de Defensa Civil Valle del Río Apurímac* - was established in Pichiwillca on the 21st of June of 1984. In the following years, the committee of Pichiwillca became the driving force behind the proliferation of the model of self-defense throughout the entire valley by obliging peasants in every single locality to organize themselves in what they called *Comités de Defensa Civil Antisubversiva* (DECAS) (CVR, 2003, p. 83). Every new-founded *comité de base* corresponded to a geographical *sector* and was steered by a board of directors, consisting mostly of a president, vice-president, secretary of acts, treasurer, operations commander and intelligence officer (Fumerton, 2002, p. 116). The overall organizational structure was highly hierarchical: the headquarter in Pichiwillca instructed the so-called *comités zonales* which on their turn instructed the *comités de base* (Del Pino, 1996, p. 153; Fumerton, 2002, p. 142). By 1993, 393 communities had organized in base committees and twenty-three zonal committees existed.^8^

Despite Shining Path’s brutal responses to the peasants’ resistance, the self-defense committees proved efficient in regaining control over the territory. By the end of 1989, the CADs succeeded in controlling the entire valley, from Anchihuay in the South to Boca Mantaro in the North (Fumerton, 2002, 142). While 1984 to 1986 were the bloodiest years, the war in the valley raged until the mid-nineties. As Percy, an ex-CAD commander in the district of Llochegua, recounts: ‘Between 1983 and 1995, there were no laws, guns were the masters of life’ (author’s interview, 10 May 2017). The peasants’ war against Shining Path came, however, at a high price. Several research participants in the districts of Santa Rosa, Kimbiri and Llochegua mentioned the excessive use of violence by the CADs and their involvement in personal reprisals and struggles over power. Over the span of the entire armed conflict, armed civilians were involved in 409 of the 8,202 violent incidents in the Ayacucho department registered by the CVR (Zech, 2016, 83).

Although the self-defense committees originally emerged from spontaneous peasant action, the marine forces facilitated and, in some places, even enforced their organization under the name of *Comités de Defensa Civil* (CVC) (Del Pino, 1996, p. 139). In 1991, under the first government of president Alberto Fujimori, the self-defense committees were legalized under the name *Comités de Autodefensa* (CAD), and allowed to use firearms ‘obtained by purchase or by donation from the State or individuals’ in ‘activities of self-defense of their community to avoid infiltration of terrorists and drug traffickers, to defend themselves from their attacks and to help the Peruvian Army and the National Police with the tasks of pacification and national development’ (DL 741, 1991).^9^ This legalization served to gain stricter control over the CADs in order to prevent them from transforming into independent death squads or criminal organizations (Fumerton, 2018, p. 80).

The alliance between the CADs and the state forces, which became stronger from the beginning of the 1990s, was complex and volatile throughout the whole course of the war. In first instance, collaborating was a pragmatic choice for both parties. Due to the strong repression, the peasants in many cases perceived the state forces as a greater threat than Shining Path. By consolidating their alliance with the military, the peasants demonstrated which side they were on and liberated themselves of the constant suspicion of pertaining to Shining path (Fumerton, 2002, p. 114). Furthermore, settlements reluctant to organize in self-defense committees risked brutal punishment by the military or by notorious CADs, such as those of Pichiwillca (Fumerton, 2002, p. 117). The marines and the military, in turn, were unfamiliar with the geography of the valley and Shining Path’s guerrilla warfare, and therefore needed the CADs’ field knowledge. Initially there was, however, a lot of mutual distrust, as Alfonso ex-commander in the district of Pichari, testifies:

They did not believe us [when we said that the Shining Path was present], it was difficult to establish this relation with the army [...] we needed the army for their fire force, not because they were so brave [...] Step by step, we entered in a relation and in the end, they gave us this confidence and then we have cooperated well [...] but the biggest part, the biggest part, almost 90% of what has been done was the self-defense, that is, the peasant population. (Author’s interview, 10 July 2018)

Notwithstanding their alliance with the military, the CADs maintained a high degree of autonomy and controlled most of the valley, as a result of which they became the main target of Shining Path’s attacks (CVR 2003, p. 84). Violent confrontations occasionally took place between the CADs and the army as well, for example, when the latter entered a CAD-controlled zone without permission (Del Pino, 1996, p. 169). The relation between the CADs and the police — notorious for siding with the big landowners and discriminating against the peasant population — was (and in many places remains) outspokenly bad.

The escalation of the internal armed conflict furthermore coincided with a deep economic crisis caused by increasing insecurity and the rise of illicit drug trafficking in the valley. Peasants’ restrained access to lands and markets caused the collapse of traditional crops, leading to scarcity and famine. The increasing international demand for coca leaves for the production of cocaine paste and cocaine formed an incentive for peasants to focus on cultivating coca leaves; a crop which is both less time-consuming and more profitable, and can moreover be harvested year-round. In addition, the government’s counterinsurgency strategy became catastrophic during the presidency of Alan García (1985–1990), resulting in regular mutinies within the army. The CADs were hence in need of new allies in their struggle to control the valley (Fumerton, 2002, p. 120), which is how a pragmatic alliance between the self-defense committees — who made a living as *cocaleros* — and drug traffickers *(narcotraficantes)* arose between 1985 and 1987 (Del Pino, 1996, p. 127). The CADs shielded the *narcotraficantes* from Shining Path incursions while the *narcotraficantes* — initially mostly connected to Colombian drug cartels — assured the demand for coca and provided arms, money and food (Del Pino, 1996, p. 167). The economic surplus generated by the coca boom allowed the CADs to form the *comandos especiales*, the so-called Tigers and Condors: elite militias who received professional training and a monthly salary (Fumerton, 2002, p. 142). The disruption of the Colombian cartels after the death of Pablo Escobar in 1993 and the repressive anti-drug policies under the government of president Fujimori caused a sudden collapse of the drug economy in 1995, but by the end of the decade, the region had recovered from the crisis and returned to the production of coca for the drug market (Heuser, 2017, 200–201). Until today, illegal coca crops remain the principal source of income for most peasants in the region.

Notwithstanding the illicit economy, the VRAEM is one of Peru’s poorest regions. Only one out of two inhabitants of the VRAEM have direct access to basic health care, and education levels are far below the national average, with 29% of youngsters between the age of 20 and 24 not having completed primary education (INEI cited in: Heuser, 2017, p. 184). Furthermore, the VRAEM is still a militarized emergency zone where the Peruvian army and the police are fighting a war on drugs against the so-called *narcoterroristas*, remnants of Shining Path now involved in drug trafficking. Ironically, the presence of the state forces seems to increase rather than decrease the population’s feeling of insecurity, because they are generally perceived as corrupt and incompetent (Heuser, 2017, 228; Willems, 2020). This negative perception of the state and its representatives is one of the reasons why the CADs are still important providers of authority, order and security for the population in the valley.

However, while the self-defense committees were never demobilized nor disarmed, their role has changed significantly since the end of the internal armed conflict. Participation is no longer mandatory and, in most villages, the CADs mainly function as a local security force handling daily crimes such as robbery and domestic violence, intervening in skirmishes during festivities, or providing general order during moments of crisis. During the covid-19 pandemic, for example, the CADs controlled the flow of incoming and outgoing travelers as well as the compliance with quarantine and other lockdown measures to prevent the spread of the coronavirus. The legitimacy of the self-defense committees today relies in large parts on the heroic collective memory of their successful struggle against Shining Path in the 1980 and 1990s, which is celebrated every year on the 21st of June during the commemoration ceremony of the foundation of the central self-defense committee of Pichiwillca. Throughout the valley, the self-defense committees and their mythical leaders are honored in public space, such as for example in the towns of Santa Rosa and Palmapampa, where a statue of Antonio Cárdenas stands on the main square. In June 2022, a new law was approved that reaffirms the self-defense committees’ permission to carry arms. It moreover explicitly states that the CADs operating in emergency zones (i.e., the VRAEM region) ‘realize activities of self-defense’ and ‘support the armed forces and the National Police of Peru in the struggle against illegal drug trafficking and terrorism in the framework of national pacification efforts’.^10^ The law’s approval did not pass without public controversy, as critics argue that it diametrically opposes earlier intentions to demobilize the CADs, and opens the door for peasant communities throughout the country to organize in self-defense committees and take up arms, with all its consequences (La Mula, 2022).

## Becoming a combatant, a macho and an adult

To understand youngsters’ motivations to organize and participate in armed resistance against Shining Path, it can first of all be insightful to have a closer look at the overall strategies of organization of the CADs. To begin with, youngsters were a considerable part of those who were (forcibly) recruited to participate in self-defense: per family at least one healthy man or woman between the age of more or less fifteen and seventy years old was obliged to join the CADs. While parents often objected the recruitment of their children, only the few youngsters who were studying in high school were generally exempted from participating. Ladislao, an ex-CAD member in the district of Samugari, recounted, for example, how his mother wanted him to attend school rather than to join the CADs. When the self-defense committees came to recruit him, his mother repeatedly succeeded in preventing this by arguing that her son was studying:

On one of these occasions, they started recruiting [*sacar*] me as a *patrulla*, and my mother came, she cried and grabbed me and said: “My son is a student, how are you going to do this to him, how is this possible?”. And many of them said: “Let that boy go, he is a student”, and I came home. (Author’s interview, 24 June 2018)

Ladislao himself, however, says that he was tempted to join the CADs: ‘I wanted to go, I wanted to know the reality elsewhere *[conocer la realidad en otra parte]’*. Besides being prone to adventure, Ladislao also seemed to have a motive of vengeance to join the CADs. His mother was being sexually abused by one of the village authorities and, as a result, gave birth to two children, Ladislao’s little brother and sister. When describing why, eventually, at the age of sixteen, he decided to join the CADs voluntarily, Ladislao stated:

I wanted to take revenge on that dick *[cojudo]*. How was he going to do all of this to my mom? That was the reason why I decided not to hesitate anymore and join the *patrullas* [CADs] straight away. I told my mom one day and when she cried, I said: “Don’t judge me ma, I am a big boy now *[ya soy mayorcito]*, I have to go”. (Author’s interview, 24 June 2018)

The fact that Ladislao’s mother was a single parent moreover stimulated him in the conviction that he had to take up his role as a male head of the family and protect his mother and his siblings:

I was a real man already *[ya era macho ya yo]* and from then on, I had other goals. I told my mom: “Leave me, ma, I am not going to study, I will find myself a partner, and I want you to get out of this difficult situation, I am the father now”. (Author’s interview, 24 June 2018)

Participation in the CADs involved duties directly related to self-defense including patrolling, but communal work such as cleaning irrigation canals and repairing roads was also organized and executed by the self-defense committees. While both men and women took part, they were assigned different tasks which were mostly gendered, with women for example preparing the food for the patrols, or participating in vigilance but not in combat. Nevertheless, when the village was under attack, men and women alike were expected to take part in the community’s defense. In some communities, women took up leading roles and formed separate women’s commandos *[comando de mujeres]*. Flora, whose parents were both prominent self-defense leaders in the district of Santa Rosa, narrated how her mother eventually committed suicide as a result of the constant persecution and intimidation by Shining Path. As a young girl, Flora also participated in self-defense herself:

All the girls did surveillance. We all participated. We even gave weapons to the children; the children made their weapons out of sticks. So many children said: “Look, I am Huayhuaco *[a notorious self-defense commander]*.” (Author’s interview, 11 July 2018)

Disobedience was not tolerated and participation in the self-defense committees was enforced through fines and corporal punishments such as whipping (Fumerton, 2002, p. 70). The CADs moreover had their own intelligence service and were constantly on the lookout for infiltrated Shining Path members, which for example resulted in violent nightly house-to-house searches (Del Pino, 1996, p. 148). Shining Path members who were captured by or defected to the self-defense committees were given the chance to switch sides through the practice of *arrepentimiento*: the act of making a statement of remorse which in some cases went together with corporal punishments. Ivan, ex-CAD member in the district of Samugari, describes the process as follows:

For example, there were always persons who met *[arrepentidos]* while they were working on their land, so they brought them to the self-defense, and they informed them that they had found an *arrepentido.* And then the *arrepentidos* confessed: “I have come from that place, and this is how things were, and I could not bear it any longer, and that is why I have left.” […] And the self-defense forgave them. (Author’s interview, 27 June 2018)

The *arrepentidos* were mostly reintegrated into the community and forced to participate in self-defense, as they could provide useful information about the enemy and its strategies. Many of these *arrepentidos* were children and youngsters forcibly recruited by Shining Path, forgivingly referred to by some research participants as *mocosos* [snot-nosed children], as Oliver, an ex-CAD commander in the district of Pichari recalls:

They [the *arrepentidos*] were humble peasants, poor *mocositos*. They were recruited. What were they to blame? So, we could not kill them either. The only thing we had left to do was to try to rescue them. (Author’s interview, 9 July 2018)

Indeed, forced recruitment was a systematic practice of all armed actors involved in the internal armed conflict, and nearly 40% of all victims of forced recruitment were minors. Apart from youngsters’ voluntary and forced participation in self-defense committees, Shining Path and the state forces kidnapped and forcibly recruited minors as messengers, spies, fighters and sex slaves (Herencia Carrasco, 2010, p. 8). This means that youngsters’ participation on either side of the conflict — whether as part of self-defense committees, insurgent groups or state forces — should not necessarily be aligned with a fixed ideological or political position. 11

The trajectory of Javier Pompeyo Rivera, aka *comandante* ‘Huayhuaco’, is a telling example of civilians’ alternating positions during the internal armed conflict. While Huayhuaco himself always denied any affiliation with Shining Path (Fumerton, 2002, p. 126), according to historian Ponciano Del Pino and as confirmed by one of my research participants, he was an *arrepentido* who, after having served a prison sentence in Lima for his involvement in drug trafficking, returned to the valley and took up the leadership of the self-defense committee in the town of Rinconada in 1988 (Del Pino, 1996, p. 149; Fumerton, 2002, p. 125; author’s fieldnotes, 26 June 2018). Before becoming a drug trafficker, a Shining Path rebel and subsequently a self-defense commander, Rivera had been both a primary school teacher and a dentist, offering, as the anecdote goes, tooth crowns with a hammer and sickle to Shining Path *camaradas* visiting his dental practice (author’s fieldnotes, 26 June 2018; Fumerton, 2002, p. 126). According to a news article published by IPS in 1996, Huayhuaco would have engaged in self-defense because he wanted to take revenge for the murder of his wife (IPS, 1996).^12^ Together with Antonio Cárdenas, he became the key figure behind the organization and proliferation of the CADs in the Apurímac valley in the late 1980s. Inspiring a mixture of admiration and fear, both Huayhuaco and Cárdenas are generally remembered as natural leaders with an extraordinary talent for organization and military strategy, while at the same time being notorious for their excessive use of violence against (presumed) Shining Path members and their ruthlessness against the local population in case of disobedience.

While Huayhuaco was in his early forties when he became a self-defense commander after having gone through a diverse trajectory of diverging occupations and affiliations, Antonio Cárdenas was only nineteen years old when he founded the self-defense committee in the town of Pichiwillca in 1984 and became the president of the CADs of the entire Apurímac valley. According to Huayhuaco, Cárdenas was young, unprepared and lacking the necessary education when assuming the presidency, which is why he would have nominated Huayhuaco to become the general coordinator of the self-defense committees’ military operations (Fumerton, 2002, p. 124). Huayhuaco’s account of the motivations behind organizing self-defense expresses a pragmatic strategy of survival rather than a purely ideological stance against Shining Path’s revolutionary struggle: he believed that an alliance between civilians and the military was the best option to prevent the peasants from being constantly targeted by the state forces on the suspicion of belonging to Shining Path, and would hence ‘enable pacification to be achieved with a minimum costs of lives’ (Huayhuaco 1991 cited in Fumerton, 2002, p. 130). Huayhuaco’s central role in the CADs was however short-lived: in 1990 he was arrested once more for involvement in drug trafficking and became *persona non grata* among the self-defense leaders (Del Pino, 1996, p. 151). He currently lives in Argentina (author’s fieldnotes, 11 July 2018).

Apart from *arrepentidos* like Huayhuaco and many others, youngsters who were forcibly displaced from their home regions because of the general insecurity and the economic hardship caused by the war also joined the self-defense committees in the Apurímac valley as a means of protection and survival. One of the documents found in the archive of the CADs in Pichiwillca, for example, consists of a declaration signed by five young men between the age of fourteen and twenty-one, who state that they have come to join the local self-defense committee ‘on a voluntary basis […] as we don’t have food let alone work in our place of origin’.^13^ Indeed, for many youngsters, participation in armed groups was in the first place a necessity for survival and reflected enforced or pragmatic decision-making processes.

In addition, gendered ideas on the respective roles that men and women should assume during armed conflict seem to have formed an incentive for young men to join the self-defense committees, as is demonstrated by the trajectory of Ladislao described above: he joined the CADs to protect his mother and siblings and to avenge the man who raped his mother. The idea that men have to protect their family is a central element of the masculine values associated with *machismo*, which in this context can be related to the organization of self-defense. Ladislao’s emphasis on the fact that he had become a ‘*macho’* and a ‘father’ reflects his desire of being perceived as a real man, obtaining adult status, and leaving his childhood behind (Bourdieu, 1993, p. 96). The process of becoming a CAD combatant is hence closely connected to the process of entering adulthood and embracing certain roles and values associated with masculinity. Such masculine values stood center stage in the militarization of daily life during the internal armed conflict. The commanders who made up the board of directors of the CADs were often *licenciados* (veterans of the Peruvian army) fostering a warrior identity shaped at the intersection of militarization and *machismo*.

Throughout the war, this *macho* warrior identity was progressively constructed as activities related to self-defense became a priority while normal daily routines disappeared to the background. As Eduardo, an ex-CAD commander in the district of Pichiwillca who joined the self-defense militias at the age of sixteen, affirms: ‘Between 1983 and 1995 we dedicated ourselves fully to *la lucha* [the struggle], nobody was getting married or building their houses’ (author’s interview, 1 October 2021). As communities were labelled “bases” and peasants became soldiers, ‘military life came to the valley’, as Francisco, an ex-CAD commander in the district of Samugari puts it (author’s interview, 24 June 2018). Values such as discipline, obedience, strength and loyalty became central elements of the organization of daily life. The nicknames of the commanders, such as Beast or Dragon, represented their military alter ego. Both the fact that many of the commanders were veterans, and the frequent alliances with the Peruvian army, contributed to the construction of this military identity.

Moreover, through their emblematical leadership, commanders such as Huayhuaco or Antonio Cárdenas became mythical figures starring the heroic narratives of pride and resistance which played a key role in mobilizing peasants to engage in self-defense (Zech, 2016, p. 107). The construction of masculine heroes and living legends thus became one of the many strategies of survival and resistance, and for many youngsters participating in self-defense meant a coming of age as a combatant, a *macho* and an adult in the midst of war. As Degregori (1991) similarly observed when looking at the motivations of young rural Shining Path members seduced by the promise of action and prestige, joining an armed group for some youngsters became a rite of passage.

## Ex-combatants’ claims for recognition, reparation and citizenship

For an entire generation of CAD commanders and combatants, the war was indeed their primary formative experience, as they were only young men when Shining Path took up arms and they decided to resist. As Kimberly Theidon states about the Colombian ex-combatants she conducted research with, ‘these men embody their violent pasts in enduring, albeit often unconscious, ways’ (Degregori, 1991). For those who participated in armed combat, the psychological experience of the war is inevitably interrelated with the physical state of their body which has been trained for, and in many cases mutilated by, the war. Rafael an ex-commander in the district of Pichari, suffers severe health consequences as a result of his time as a CAD commander:

I have broken three ribs, regular stuff, but I survived. I have ammunition here, and here [points at body]. I have seventeen scraps in my entire body. A bullet in my leg. That’s why I limp a little. […] I lost my sight, they blew me up here, all of this [points at body]. (Author’s interview, 12 May 2017)

Many of the ex-commanders and members of the self-defense committees moreover indicate that they suffer from mental health problems and that this impacts their social relations. According to ex-CAD commander Percy, ‘the VRAEM needs mental healthcare to restore confidence. We have seen terrible things.’ (Author’s interview, 10 May 2017) Humberto, who was a CAD commander in the district of Kimbiri, tells that he sometimes suffers from violent anger attacks because of what he has lived through (author’s interview, 23 June 2018). Among other psychosocial consequences, alcoholism is a recurring problem among ex-CAD commanders and members.

Indeed, in the VRAEM, as in other regions affected by the internal armed conflict, two decades of war have severely thwarted the personal development and future perspectives of an entire generation of youngsters. As Rafael, who is now in his early sixties, puts it:

I have spent my entire youth fighting. And now that I am injured, I am already old and injured, I don’t see well anymore. [...] I have used up my entire youth in the bush. (Author’s interview, 12 May 2017)

The way in which many ex-commanders still hold on to their warrior identity demonstrates the extent to which they were formed by the experience of the war. This identity is closely tight to certain ideas about juvenility and masculinity. Rafael for example complained about the fact that his merits as a CAD combatant are not recognized by his fellow villagers by stating that he is no longer perceived as a *macho* (author’s interview, 12 May 2017). In addition, many ex-CAD commanders still cherish their weapons — an essential part of their warrior identity — and express to be ready to use them if necessary. As Rafael affirms:

Until today I have rifles in my house. It is for my defense. [...] There are no ex [commanders] here. We are not ex, we continue. (Author’s interview, 12 May 2017)

Furthermore, the process of becoming a CAD combatant for many youngsters was not only connected to the process of entering adulthood; it also represented a means of seeking connection with the nation-state. Despite its geographical isolation, the emergence of the self-defense committees during the internal armed conflict in the Apurímac valley was not just an application of “the law of the jungle”: the struggle of the CADs was deeply embedded in their relationship with the state and their militia service became a way of claiming belonging to the nation. As Alejandro, an ex-CAD commander in the district of Pichari explains:

When we started our armed struggle, they borrowed us their weapons. [...] The marine forces said: “These weapons are from the state and you are the state. Take the weapons, a lance, a grenade, take it.” The captain gave me a box of grenades, his ten FALs [*Fusil Automatique Léger*], his mines [...] and in that way, we started to reconquer all the spaces of the villages, creating new villages, putting people there, and we reconquered all of this. (Author’s interview, 8 July 2018)

The CADs became representatives of the Peruvian state by taking up arms against Shining Path to “liberate” the local population from the revolutionaries. Weapons were not only an essential part of a *macho* warrior identity, they also turned into a means for peasant youngsters to “become Peruvians”. For a population group whose citizenship had been - and in many ways still is - historically thin due to racial discrimination and socio-economic exclusion, militia service became a foundation for claiming a thicker citizenship. The context of the internal armed conflict hence created a space for negotiation between the state and its rural populations, as the state eventually realised that it could not win the war without the CADs, and entered in ‘an alliance that exchanged support for recognition’ (García-Godos, 2005, p. 40). Demanding this recognition for their contribution to the defeat of Shining Path became an essential aspect of the CADs’ appropriation of citizenship in the aftermath of the internal armed conflict.

The hope that this recognition would become reality was sparked by the abovementioned legal framework of 1991–1992, which in the first place institutionalised the CADs as a legitimate actor in the present rather than recognising their contribution to the defeat of Shining Path during the internal armed conflict.^14^ In subsequent years, however, the expectation that the latter would also become reality was not met. A supreme decree issued in 1998 (068-98-DE-S/G) set out a framework for monetary compensations for death, disability or material damage suffered by CADs during the conflict, but proved disappointing as the criteria were so strict that as few as twenty-nine persons could benefit from it (CVR 2002, p. 48). One of the most problematic characteristics of the compensation decree was that it only issued compensations from 1992 onwards, when the CADs were officially recognised, hereby ignoring the casualties of the first and bloodiest decade of the war (García-Godos, 2005, 212). In 2001, the CVR was installed which eventually led to the implementation of a large-scale reparation program for victims of the internal armed conflict (the *Plan Integral de Reparaciones*, PIR). The PIR included CAD members injured in confrontations with Shining Path into the universe of victims – much to the dismay of many self-defense members who see themselves in the first place as heroes rather than victims (García-Godos, 2008, 79). By being forced to adopt the discourse of victimhood, the CADs felt stripped from their agency as armed civilians and defenders of the nation.

Today, the heroic memory of the CADs in the Apurímac valley is interwoven with bitterness and disillusion. Statements about the lack of recognition for their contribution run as a red thread through conversations with ex-CAD members and their relatives:

As self-defense, we went all the way, so that time, often we have fought in exchange for nothing, *señorita.* Up until now, the government doesn’t give us any recognition, we simply have this legal regulation, nothing more [...] (Author’s interview with Sebastián, ex-CAD member in the district of Santa Rosa, 27 June 2018)

In fact, [we brought about] the pacification in the VRAEM, that is the truth. The army they did little or nothing, maybe they served as a shield, but who was the cannon fodder? Who has marched? Who has captured? It was us, complying their duties [...] Honestly, until this moment, it gives me a lot of sorrow. (Author’s interview with Percy, ex-CAD commander in the district of Pichari, 9 July 2018)

This sense of lack of recognition for the CAD’s past contribution goes hand in hand with the socio-economic marginalization and isolation of the region within the national context during the present day. The fact that the VRAEM is labeled as a territory in state of emergency where a war against drugs is being fought differentiates the region to a certain extent from the ‘pacified’ areas of the country. Research participants often link this sense of isolation to the poor socio-economic situation of the region, as the persistence of the emergency zone is seen as an obstruction to development and inversions in the VRAEM. Ladislao, who is also involved in local politics, stresses the importance of development and education to prevent future outbreaks of violence in the region:

The pupils have several diseases, it looks as if we cannot accomplish anything. First, we need nutrition, first we need water and basic sanitation, nutrition, if not, how are we going to talk about education? [...] So, analyzing all these things [...] the situation is disastrous, and with all the more reason any delinquency, any dirty dark politics can appear. (Author’s interview, 24 June 2018)

Ex-CAD commander Percy describes the precarious situation of many survivors and the intergenerational consequences of the internal armed conflict as follows:

We are going to return to the same problem of before because let’s say that *la subversión Sendero* [Shining Path] would have killed like twenty people, but their children, their wives, some of them widows or widowers, the husbands of whom the wives were killed, they have left orphans who did not have a good education nor any support from the government. One day, they will feel the sorrow because they have seen the dead body of their parents. So, these girls today are single moms and the boys are delinquents. [...] I know that with these problems ahead, the same situation will rise again. The people will not have anything to live from and they will dedicate themselves to assaulting, stealing, some will maybe go to other places, I don’t know, but for the majority who will stay, this conflict will not end. (Author’s interview, 9 July 2018)

In the Apurímac valley, this frustration of personal lives by the outbreak of the war is inevitably linked to the phenomenon of self-defense and the active role of armed civilians in the struggle against Shining Path. More than in other areas, daily life in the valley became militarized as peasants took up arms. An entire generation of young adults gave up their personal education to engage in the tasks of self-defense: instead of learning how to read, youngsters learned how to handle a gun. This generation of ex-CAD commanders and members now stresses the importance of education for their own children and grandchildren, hereby trying to secure the youth that was taken away from them for the future generation.

## Conclusion

During the yearly commemoration ceremony of the foundation of the central self-defense committee of Pichiwillca and homage to the leadership of Antonio Cárdenas, the so-called civil patriotic parade is the event that draws most attention of the public. Self-defense committees from the entire region send their delegations to participate in the march around the main square, displaying their guns and flags. The most remarkable participants in the march, however, are probably the school children. Nursery school toddlers dress up in the uniforms of the special CAD commandos, carry cardboard guns, and playfully swing their short legs to march like real soldiers in the footsteps of their (grand)fathers. The reenactment of the old times by the new generation serves the twofold purpose of keeping the memory of the past alive while hoping to prevent history from repeating itself.

Children and youth are generally considered among the most vulnerable in society, prone to becoming disproportionally victimized by armed conflict. At the same time, the image of the child soldier is not seldomly put forward in media and public opinion as an embodiment of the cruelest contradiction of wartime violence: children forced into adulthood by receiving guns, innocent youngsters turned warlords. The reality of youth as participants in or even drivers of wartime violence, however, requires an analysis that goes beyond the dichotomy of perpetrators vs. victims, guilt vs. innocence, adults vs. children or combatants vs. civilians. Rather than opposing all these different categories, there is a need to understand how they relate to each other and possibly overlap in the lives of those who grow up in war-thorn societies.

A closer reading of the social trajectories of (ex-)civil self-defense committee members in the Peruvian Apurímac valley first of all reveals some of the manifold reasons why youngsters decide to become active participants in wartime violence, including forced recruitment, but also the search for adventure, personal motives of vengeance, the need for protection, or the desire to become a “real man” and acquire adult status. The trajectories of the CAD members moreover clearly demonstrate how the morality of soldiering, steered by ideas about masculinity, militarism and patriotism, gets intertwined with structural societal conditions such as the lack of educational and economic perspectives for youngsters, and the state’s failure to provide protection and security against rebel group violence to those who might need it most. The self-defense committees’ practice of reintegrating former Shining Path members as CAD combatants through the process of *arrepentimiento*, for example, shows the fluidity of the ideological alignments behind youngsters’ participation in armed conflict. Simultaneously, the way in which the CADs receive the young Shining Path recruits as ‘poor snot-nosed children’ who deserve a second chance demonstrates how they consider youthfulness as a marker of innocence. It is hence this entanglement of moral and pragmatic incentives that needs to be understood to gain insight into youngsters’ decisions to take up arms.

Studying the social trajectories of (ex-)CAD leaders and members thus sheds light on their past, present and future motivations, actions and desires and the ways in which these are embedded in the socio-economic context. A coming of age at the frontline of armed conflict comes with many ambiguities that mark the lives of an entire generation of youngsters. Rather than a mere chronological order, generation can best be understood here as a social and political category. Indeed, the process of becoming a CAD combatant for many youngsters did not only reflect the desire to join in with the adults. Soldiering also became a means of claiming belonging to the nation-state and “becoming Peruvians”. This claim connects the past and the present: in the aftermath, demanding recognition for the contribution to the defeat of Shining Path became an essential aspect of the CADs’ appropriation of citizenship. As such, militia service and the corresponding *macho* warrior identity formed a basis for demanding inclusion of an historically marginalized rural population group. Today, the CADs’ general feeling that this recognition for their past merits is lacking, is reinforced by the ongoing socio-economic hardship that marks both the present-day reality and the aspirations for the future generation in the Apurímac valley.

The findings on the Peruvian self-defense committees presented in this article have several implications for research and policy in the fields of TJ and DDR, and open both thematic and conceptual avenues for further research into civilian participation in armed conflicts around the globe. Firstly, on an analytical level, dismantling the victim-perpetrator binary by studying civilian participation in armed conflict allows us to sketch a more complex image of certain conflict actors and the motivations that shape their actions, as well as how these might change over time. Looking at structural conditions of socio-economic exclusion moreover adds a layer of temporality to the analysis that is often lacking, but is crucial to understand claims for recognition, reparation and citizenship formulated by survivors of armed conflict. Furthermore, the findings presented in this article generate insights into how notions of heroism and masculinity shape processes of meaning-making, collective memory, and identity construction among civilians who participate(d) in combat. The fact that discourses that fit the language of militarism rather than that of peace and human rights are deployed by survivors to formulate claims for recognition, reparation and citizenship that align with TJ and DDR efforts, constitutes a paradox which once again shows that the complexity of (post-)conflict actors and landscapes cannot be grasped through a dichotomous framework. In practice, transcending the victim-perpetrator binary and making more space for this complexity might also contribute to bridging the gap between TJ and DDR efforts, and facilitate a better coordination between policies that are being developed in the respective areas.

## Data Availability

The datasets generated during and/or analysed during the current study are not publicly available due to privacy reasons but are available from the corresponding author on reasonable request.

## Footnotes

1 Hereafter: Apurímac valley.

2 Shining Path emerged as a radical splinter group of the Red Flag Maoist Communist Party of Peru at the university of Ayacucho in the 1970s and initiated an armed struggle with the aim of overthrowing the Peruvian government in May 1980. Starting from 1982, the state forces intervened and the situation escalated into a complicated civil conflict marked by multidirectional violence. According to the Peruvian Truth and Reconciliation Commission, approximately 69.000 Peruvians were killed or disappeared between 1980 and 2000, with Shining Path being accountable for 54% of the casualties and the state forces 37%.

3 According to Zech, armed civilians were involved in 409 of the 8.202 violent incidents in the Ayacucho department during the internal armed conflict registered by the TRC (Zech, 2016, p. 83).

4 The sources from this archive cited in the present article are referred to with the abbreviation “ACADVRAE”.

5 *Barbasco* is a plant containing strong chemical compounds that was formerly used as an organic insecticide.

6 Small farm, the opposite of a hacienda.

7 The organization of civil self-defense committees as it came about in the Apurímac valley shared characteristics with a tradition that already existed in other parts of the country before the internal armed conflict. In the 1970s, peasants organized in *rondas campesinas* in the Northern Andes region of Cajamarca to protect their communities from cattle rustling and other common crimes in a region where the presence of the state was poor or non-existent. For a detailed account of the phenomenon of the *rondas campesinas* in the Northern Peruvian Andes, see: Starn (1999). When the self-defense committees in the VRAEM emerged, there was confusion between them and the phenomenon of the *rondas campesinas* from the North, and the terms *rondas campesinas* and *comités de autodefensa* were often used interchangeably. The *rondas campesinas*, however, distanced themselves from the self-defense committees in the VRAEM and their use of violence. In a document issued by the NGO SER in 1986, a comparison is made between the *rondas campesinas* and the self-defense committees, which mainly depicts the *rondas* as a peaceful organisation and the self-defense committees as a violent militia (Burneo Labrín & Eyde, 1986).

8 ACADVRAE, Breve reseña histórica, 1993, p. 6.

9 Decreto Legislativo 741 (1991), ‘Reconocen a Comités de Autodefensa, como organizaciones de la población para desarrollar actividades de auto defensa de su comunidad’, https://www.leyes.congreso.gob.pe/Documentos/DecretosLegislativos/00741.pdf. This does not mean that firearms were not used before this legislation. Many peasants who did not have the resources to buy firearms fought, however, with bladed weapons. Interestingly, Zech argues that ‘weapons and other resources make self-defense forces more capable but are not a necessary condition for sustained civilian resistance. Organisational capacity and technical expertise played a more important role’ (Zech, 2016, p. 233).

10 Ley n°31,494 (2002), ‘Ley que reconoce a los comités de autodefensa y Desarrollo rural y los incorpora en el Sistema de seguridad ciudadana’, https://busquedas.elperuano.pe/normaslegales/ley-que-reconoce-a-los-comites-de-autodefensa-y-desarrollo-r-ley-n-31494-2077948-2/.

11 On youngsters’ motivations for participation in Shining Path in the city of Ayacucho, see Walker (2022). In other work on peasants’ support for and resistance against Shining Path in rural communities in Ayacucho, I have also highlighted these shifting positions. See: Willems (2022).

12 The article does not mention who would have been the perpetrators of the killing.

13 ACADVRAE, Constancia para permanecer en el CDC, 1985.

14 Paragraph 16 of the 2022 Law n°31,494 is entitled ‘recognition’ but interestingly only speaks of the contributions of the CADs to the struggle against ‘narcoterrorism’ in the present, without mentioning their contributions to the defeat of Shining Path during the internal armed conflict. Since the fieldwork for the present article was conducted before 2022, I do not have any data on the reception of the new legal framework among the CADs.
